# Private Kindergarten Teachers' Intention to Remain: A Comparison Between the Effects of Organizational and Individual Psychological Factors

**DOI:** 10.3389/fpsyg.2022.912608

**Published:** 2022-07-08

**Authors:** Song Shi, Zizai Zhang, Hotang Wu, Xiaomei Zhang

**Affiliations:** ^1^College of Education Science, Nantong University, Nantong, China; ^2^Hangzhou Preschool Teachers College, Zhejiang Normal University, Hangzhou, China; ^3^College of Education, National Kaohsiung Normal University, Kaohsiung City, Taiwan; ^4^The School of Humanities, Arts and Education, Shandong Xiehe University, Jinan, China

**Keywords:** kindergarten teacher, private kindergarten, Intention to Remain, organizational factors, psychological factors

## Abstract

The purpose of this study was 2-fold: to validate the Private Kindergarten Teachers' Intention of Remaining (PKTIR) Questionnaire and the Factors of Teachers' Intention of Remaining (FTIR) Questionnaire, and to comparative study to explore the effects of organizational and individual psychological factors in China. A total of 22 experts were recruited to modify and validate both questionnaires. The results showed that both questionnaires had instruments that are reliable and valid. Then, a total of 582 kindergarten teachers were recruited to explore the comparison between the effects of organizational and individual psychological factors. The results from the structural equation model suggested that the influence of individual psychological factors on kindergarten teachers' intention of remaining was greater compared to the organizational factors. Second, among all the factors, the most explanatory are work connections, sense of work gained from it, satisfaction with the salary, and work discard. To our knowledge, this is the first comparative study to explore the effects of organizational and individual psychological factors in China.

## Introduction

Kindergarten teachers are important human resources for the sustainable development of kindergartens as they play a vital role in preschool education and childcare services for parents. Therefore, the quality of kindergarten teachers affects both preschool education and children's development (Gable et al., 2007). However, current kindergarten teachers have a low Intention of Remaining (Liang, 2015) and they contribute to a high turnover rate and attrition (Liu and Wang, 2014; Chen and Yu, 2017). It is obvious that the problem of how to retain kindergarten teachers is in kindergarten management. Statistics from MOE of PRC (2017) show that there is a shortage of kindergarten teachers nowadays, and Pan et al. (2017) have made an estimate for it: by 2021, kindergartens will need 3,833,900 full-time teachers and 1,916,900 caregivers, with a total demand of 5,750,800. From this, it can be judged that the shortage of kindergarten teachers is serious at present and will remain a problem in the coming years. Facing this situation, and a high turnover rate, will be detrimental to the future development of preschool education.

Private kindergartens accounted for 62.16% of preschools in 2018 (MOE of PRC, 2019). They have a shortage of teachers and a high turnover rate; many teachers do not have Teacher certificates and have low professional quality, wages, and welfare, and no protection of rights and interests and social security (Wang et al., 2015). One of the most prominent problems, which has a greater impact on the current development of private kindergartens, is the poor stability of teachers (Guo and Xu, 2018). Therefore, there is a need for an in-depth study on the private kindergartens' teachers' Intention of Remaining.

Furthermore, the quits will create high costs and burdens on the human and material resources, both for the employees and for the schools they work for (Sass et al., 2012). For example, new hires, their training, etc. will cost more money to schools (Barnes et al., 2007). Besides financial losses, the quits will also affect the educational quality (Yesil Dagli, 2012), student achievement (Goldhaber and Cowan, 2014), and the implementation of a complete school curriculum (Guin, 2004).

The results of previous studies show that many factors affect the teachers' Intention of Remaining. The focus of foreign studies on teacher turnover has gone through a process from individual to organization (Xu, 2008), with early studies on the impact of demographic variables on teachers' turnover, including gender, race, age, marital status, newborns, and a number of children in a family (Borman and Dowling, 2008). And recent researches aim at an organization or job-related factors such as work stress, salary level, social status, performance, appraisal pressure, work environment, school atmosphere, breaks and vacations, job security, professional recognition, working conditions, and job satisfaction (McCarthy et al., 2010; Liu and Onwuegbuzie, 2012; Lanas, 2017).

Teachers' turnover is primarily characterized by attrition, namely job-hopping to other schools (Boe et al., 2008). The opposite of turnover is remaining. The Intention of Remaining includes both—behaviors before turnover and psychological tendency, which expresses employees' likes and dislikes, and perceptions of their current and future jobs (Huang and Yen, 2014; Lin and Hwang, 2016). Based on the available studies, salary and work stress are two important factors for the turnover of kindergarten teachers (Kelly, 2004; Du et al., 2013; Li et al., 2015; Duan, 2017; Niu, 2017), but less mentioned in studies related to the factors of remaining. It can be seen that as the research perspectives are different, though there are common factors for turnover and remaining, specific factors are different. The factors for the teachers' Intention of Remaining can be roughly divided into three categories: demographic variable factors (Johnsrud and Rosser, 2002), such as gender, age, marital status, etc.; organizational factors (Wang et al., 2014; Grant et al., 2019), including management model and work environment; and individual psychological factors (Lin and Hwang, 2016), such as satisfaction with the salary, sense of gain from work, and work discard. During the COVID-19 pandemic, private kindergarten teachers' turnover rate decreased substantially and their Intention of Remaining was in a high position (Li, 2021). The studies on the above factors of Intention of Remaining are based on a certain perspective or certain variables, and there are no relevant studies on the systematic comparison. There are inconsistent findings on whether demographic variables significantly affect remaining in the current workplace (Chapman, 1983; Zhang and Zeller, 2016; Jiang, 2017). Based on the statistical strategy of excluding interference advocated by Chiou (2010), to fill this gap, this paper considers demographic variables as control variables to compare the impact of organizational and individual psychological factors on the Intention of Remaining.

Based on the above evidence, the aim of the present research was 2-fold: first, to develop and validate two instruments, i.e., the Private Kindergarten Teachers' Intention of Remaining (PKTIR) questionnaire and Influencing Factors of Private Kindergarten Teachers' Intention of Remaining questionnaire. Second, to include the control variables (demographic variables), organizational factors, and individual psychological factors in one model, i.e., to compare the impact of organizational and individual psychological factors on the Intention of Remaining after controlling the impact of demographic variables.

## Methods

The study consisted of two parts: the modification and validation of questionnaires; a comparison of the impact of organizational factors and individual psychological factors through questionnaires.

### Participants and Procedure

#### Participants and Procedure for Questionnaire Modification

The drafts of both questionnaires were prepared based on the existing literature. Twelve teachers from research institutes and universities were invited to revise the content of the questionnaires. Afterward, 12 additional teachers from different kindergartens, research institutes, and universities made the final modification and assessed the validity of the questionnaires. Among these experts, there were five professors, seven associate professors, three preschool researchers, five private kindergarten principals, and three private kindergarten teachers.

#### Participants and Procedure for Models

A total of 832 participants were recruited from E city, Guangdong Province. The urban area of E city was further divided into four districts and two districts with higher populations were selected for questionnaire distribution: District F with 33 private kindergartens and 848 teachers; District G with 12 kindergartens and 213 teachers. Online questionnaires were distributed to each kindergarten in the two districts. A total of 832 questionnaires were returned and 250 non-private preschools were excluded from the analysis, leaving a valid sample of 582, the 582 (100.0%) were full-time teachers, of which 302 (51.9%) were teachers with 1–5 service years, 128 (22.0%) with 6–10, 62 (10.7%) with 11–15, 49 (8.4%) with 16–20, and 41 (7.0%) with ≥ 21 service years (7.0%). Marriage status: 287 (49.3%) for unmarried teachers, 286 (49.1%) for married, and 9 (1.5%) for others (widowed, divorced); 421 (72.3%) with and 161 (27.7%) without Teacher certificates; 521(89.5%) with and 61(10.5%) without professional education background.

### Measures

#### Private Kindergarten Teachers' Intention of Remaining Questionnaire

The PKTIR questionnaire was compiled by Shi (2019), and the questionnaire consisted of eight questions. The questionnaire was scored on a Likert 5-point scale, and assigned a value of 1–5 from “strongly disagree” to “strongly agree.” After EFA, one factor was formed with commonality between 0.67 and 0.90, factor loading between 0.82 and 0.95, explained variance of 77.98%, and reliability of 0.95.

#### Factors of Teachers' Intention of Remaining Questionnaire

Factors of Teachers' Intention of Remaining in private kindergarten questionnaire (FTIR) was adapted from the I-CVI (Shi, 2019). The I-CVI was used to calculate the content validity of the questionnaires quantitatively, and if I-CVI > 0.79, the item is relevant (Zamanzadeh et al., 2015; Rodrigues et al., 2017). The adapted questionnaire included the organizational and individual psychological factors in two sub-scales with nine dimensions. The questionnaire was scored on a Likert 5-point scale, and assigned a value of 1–5 from “strongly disagree” to “strongly agree.”

Organizational Factors scale: Leadership characteristics (five items) α = 0.97, kindergarten management (nine items) α = 0.98, and work environment (11 items) α = 0.97. The final version of the Organizational Factors scale thus contained a total of three dimensions and 25 items. The commonality of the questions ranged from 0.77 to 0.93, the factor loading ranged from 0.56 to 0.85, and the total cumulative variation was 85.11%. The reliability of the 25 questions is 0.98.

Individual Psychological Factors scale: Sense of gain from work (nine items) α = 0.98, interpersonal relationships (six items) α = 0.98, work discard (five items) α = 0.97, satisfaction with the salary (three items) α = 0.84, work connection (four items) α = 0.91, and work convenience (four items) α = 0.91. The final version of the Individual Psychological Factors scale thus contained a total of six dimensions 31 items. The commonality of the questions ranged from 0.58 to 0.88, the factor loading ranged from 0.50 to 0.85, and the total cumulative variation was 77.47%. The reliability of the 31 questions is 0.98.

#### Hypothetical Model and Data Analysis

First, dichotomize the demographic variables of the formal sample. For example, teachers with 1–5 service years were coded 0, and over 6 service years were 1; unmarried teachers were 0 and married (including widowed and divorced) were 1; teachers without Teacher certificates were 0 and with Teacher certificates were 1. As there is a large gap between the numbers of gender and education background, the gender and education background variables were not included in the control variables. In addition, the monthly income variable was not included because one of the sub-scales of the questionnaire was to investigate teachers' satisfaction with the salary.

**Figure 1:**
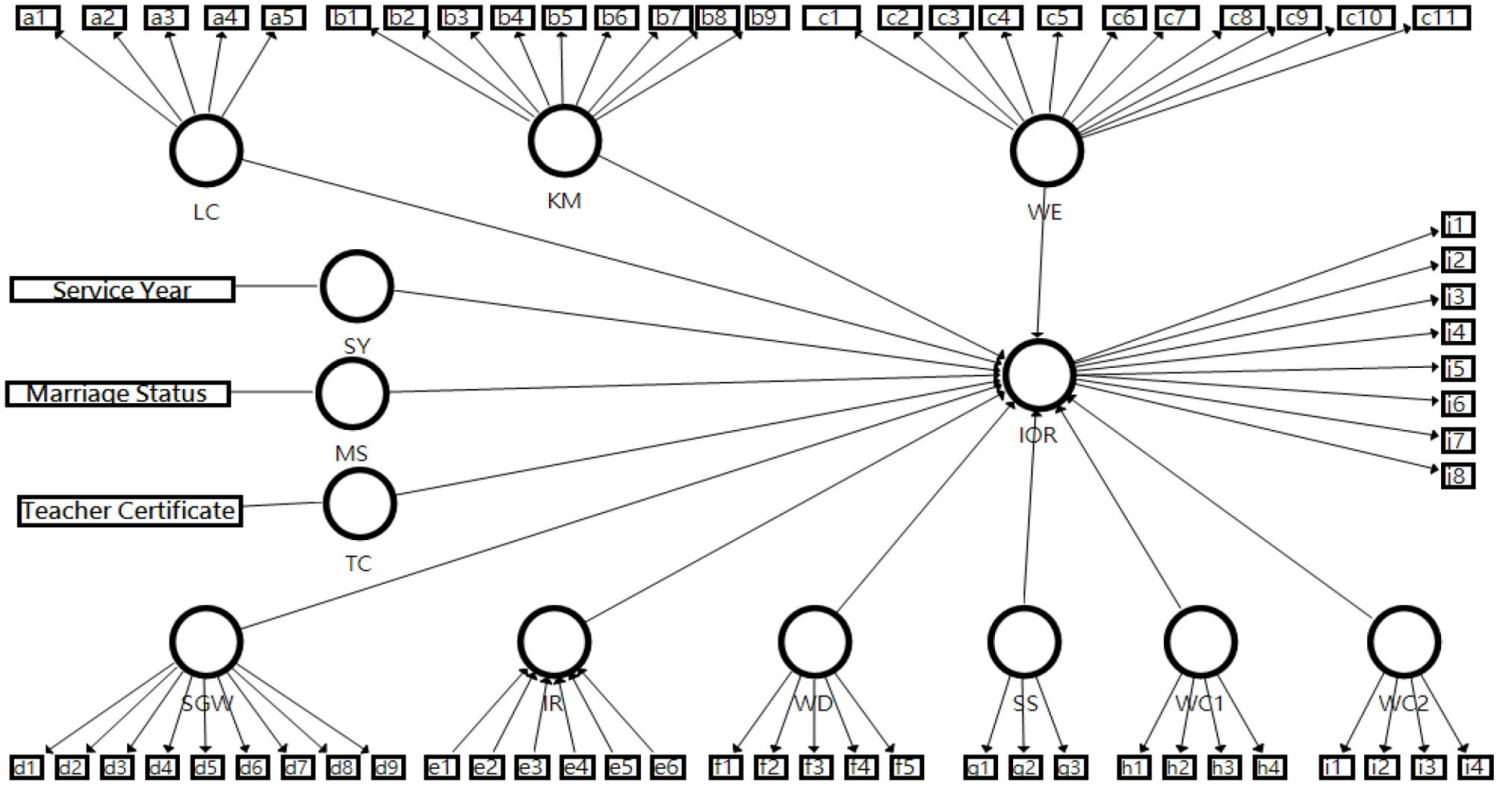
Hypothetical model.

According to the designed FTIR questionnaire, the organizational factors include three dimensions: leadership characteristics, kindergarten management, and work environment; and the individual psychological factors include six dimensions: sense of gain from work, interpersonal relationship, work discard, satisfaction with the salary, work connection, and work convenience. The above-mentioned control variables (demographic variables), organizational factors, and individual psychological factors were included in the same model (see Figure 1), i.e., the comparison of the impact of organizational and personal psychological factors on the kindergarten teachers' Intention of Remaining after controlling the demographic variables.

## Results

### Content Validity of the Two Questionnaires

Twelve experts in the field of preschool education from universities (all with Ph.D. degrees) were invited to examine the validity of the questionnaires. The Scale-Content Validity Index/Universal Agreement (S-CVI/UA) is used to determine the validity of the questionnaire. It is calculated by adding all items with I-CVI equal to 1 divided by the total number of items (Haron et al., 2019). According to the Lynn's (1986) criteria, the I-CVI value of each indicator in this study should be no <0.78, and those <0.78 show that the indicator is not much relevant to the corresponding dimension and research, and should be excluded. The results showed that the validity of all 56 questions of the FTIR questionnaire reached 0.78 or above, and should be retained. In addition, eight questions of the PKTIR questionnaires should be retained.

### CFA of the Two Questionnaires

An exploratory factor analysis was conducted on a randomly selected sample of 100 (*N* = 100). This study aimed to analyze the impact of organizational and individual factors on the Intention of Remaining; therefore, it was divided into three scales for EFA. EFA was the Varimax adopted from the principal component method and orthogonal rotation.

The results showed that the value of λ of the observed indicators for the two questionnaires with three scales ranged from 0.75 to 0.95, which met the criterion of 0.50–0.95. The higher the value of λ was, the more important the observed indicators were for potential variables to which they belonged. The higher the average variance extracted (AVE) was, the higher the convergent validity of the potential variables was, with a standard value of >0.50. Moreover, the AVE of the three scales should be between 0.54 and 0.83. Composite reliability (CR) can be considered as the inner consistency of the constructs, and the CR of the three scales ranged from 0.66 to 0.90, meeting the criterion of >0.60. Discriminant validity (DV) is the degree to which a potential variable differs from other potential variables. Fornell and Larcker (1981) asserted that as long as the square root of AVE of a potential variable was greater than the correlation with other variables, it indicated DV. In addition, the results of this study are all consistent with this condition. The validity of this study is shown in Table 1.

**Table 1:** Confirmatory factor analysis (CFA).

**latent variable**	**λ**	**CR**	**AVE**	**DV**					
				**1**	**2**	**3**	**4**	**5**	**6**
**Organizational factors**									
1. KM	0.90–0.93	0.98	0.86	**0.93**					
2. LC	0.91–0.95	0.98	0.90	0.84	**0.95**				
3. WE	0.79–0.94	0.98	0.79	0.85	0.81	**0.89**			
**Individual Psychological Factors**									
1. IR	0.92–0.95	0.98	0.87	**0.93**					
2. SGW	0.88–0.95	0.99	0.88	0.78	**0.94**				
3. SS	0.90–0.93	0.94	0.85	0.49	0.70	**0.92**			
4. WC1	0.85–0.91	0.94	0.78	0.77	0.77	0.62	**0.89**		
5. WC2	0.84–0.90	0.93	0.78	0.34	0.43	0.55	0.40	**0.88**	
6. WD	0.75–0.89	0.91	0.66	0.42	0.53	0.67	0.51	0.59	**0.81**
Intention to remain									
IOR	0.81–0.91	0.95	0.74						

*The bold black font in the DV field is the square root of AVE*.

### Common Method Variance

This study adopted the questionnaire survey method, and participants were invited to fill in two questionnaires. To avoid the common method variation (CMV), the CMV test is divided into two models: one is the CFA single-factor model, also known as the CMV model, in which all questions in the two questionnaires were classified into the same potential variable; the other is the multi-factor model, in which each question was assigned to its potential variables (dimensions) and correlation lines were set between potential variables. Analyzing the results by AMOS 22.0, χ^2^ of CFA was 20, 548.57 and the multi-factor model 56, 290.21, and Δχ^2^ = 35, 741.64 (*p* > 0.001), which reached a significant level, indicating that the CMV was not severe. Therefore, the overall model can be further analyzed.

### Overall Model

The overall model means that all influencing factors, such as demographic (control variables), organizational, and individual psychological factors, are integrated into a structural equation model. As the purpose of this study is to compare the explanatory power of organizational and individual psychological factors on the Intention of Remaining, the overall model must be fit before further comparison. The PLS is based on the number of variances, which is different from the structural equation model based on co-variation. According to Hair et al. (2006), the relevance of the model should be explained first, including the outer model and the inner model.

#### Outer Model

The outer model was based on four items to determine its relevance: indicator reliability, which was between 0.556 and 1.000 (the demographic variable had only one indicator, so the factor loading was 1.000), meeting the criterion of 0.50 or above in this hypothetical model; CR, which was between 0.847 and 1.000, meeting the criterion of 0.70 or above; AVE, which was between 0.935 and 1.000, meeting the criterion of 0.50 or above; DV, which met the criterion proposed by Fornell and Larcker (1981) that the square root of AVE of a factor was greater than the value of the correlation between the potential variable and the other variables (Table 2). In conclusion, all four items met the criteria and indicate that the measurement model is relevant. The indicators reliability, CR, AVE, and DV of the outer model (measurement model) of the overall model met the criteria (Table 3), and therefore, the outer model was relevant.

**Table 2:** Discriminant validity test results of the model.

	**1**	**2**	**3**	**4**	**5**	**6**	**7**	**8**	**9**	**10**	**11**	**12**	**13**	
1.IR	**0.93**													Y
2.LC	0.60	**0.95**												Y
3.MS	0.13	0.12	**1.00**											Y
4.WC2	0.34	0.36	0.31	**0.88**										Y
5.WD	0.41	0.40	0.06	0.59	**0.81**									Y
6.SGW	0.78	0.72	0.13	0.43	0.53	**0.94**								Y
7.WE	0.76	0.81	0.11	0.45	0.55	0.88	**0.89**							Y
8.WC1	0.77	0.58	0.08	0.40	0.51	0.77	0.75	**0.89**						Y
9.SS	0.49	0.55	0.08	0.55	0.67	0.70	0.70	0.62	**0.92**					Y
10.SY	0.10	0.06	0.68	0.24	0.09	0.12	0.08	0.07	0.12	**1.00**				Y
11.IOR	0.68	0.62	0.09	0.49	0.61	0.82	0.78	0.80	0.76	0.10	**0.86**			Y
12.KM	0.73	0.84	0.10	0.40	0.49	0.84	0.94	0.69	0.66	0.06	0.73	**0.93**		Y
13.TC	0.09	0.03	0.21	0.02	0.01	0.06	0.06	0.07	0.03	0.32	0.05	0.04	**1.00**	Y

*Diagonal bold boldface is the square root of AVE*.

**Table 3:** Reliability test results of the outer model.

	**LV**	**IR**	**CR**	**AVE**
Organizational factors	LC(a1-a5)	0.83–0.93	0.98	0.90
	KM(b1-b9)	0.85–0.90	0.98	0.86
	WE(c1-c11)	0.71–0.89	0.98	0.79
Individual psychological factors	SGW(d1-d9)	0.77–0.91	0.99	0.88
	IR(e1-e6)	0.84–0.90	0.98	0.87
	WD(f1-f5)	0.57–0.80	0.91	0.66
	SS(g1-g3)	0.81–0.87	0.94	0.85
	WC1(h1-h4)	0.73–0.80	0.94	0.78
	WC2(i1-i4)	0.71–0.82	0.93	0.78
	IOR(j1-j8)	0.66–0.90	0.96	0.74
Demographic variables	MS	1.00	1.00	1.00
	SY	1.00	1.00	1.00
	TC	1.00	1.00	1.00

#### Relevance of Inner Model

The relevance of the inner model is further assessed based on the relevance of the outer model. PLS-SEM is based on the variances, different from the covariance-based structural equation model (CB-SEM) instead of estimating the difference between the sample covariance matrix and the theoretical model covariance matrix, the model uses sample data to obtain the parameters that best predict the inner derivatives. Therefore, there are no standard relevance indicators for PLS-SEM (Hair et al., 2014). This study provides the following indicators to discuss the relevance of the inner model.

The standardized root mean square residual (SRMR) is the only indicator available to assess the relevance of the PLS path model (Hu and Bentler, 1998). The standard value of SRMR is <0.05 (Byrne, 1998) or 0.06, or a little higher (Henseler et al., 2014), while the hypothetical model in this study has an SRMR = 0.049, meeting the criteria.

Cross-validated redundancy (*Q*^2^) is obtained by PLS using blindfolding, a sample reuse technique that calculates the values of Stone-Geisser's *Q*^2^ to show the cross-validated predictive relevance. If *Q*^2^ > 0, the measure has predictive relevance to the constructs. In this model, the endogenous variable is the Intention of Remaining, and others are exogenous variables. The *Q*^2^ of all the exogenous variables on the Intention of Remaining is 0.255 > 0, indicating a predictive relevance.

The coefficient of determination (*R*^2^) is an indicator of the predictive accuracy of the model and represents the joint effect of all exogenous variables on the endogenous variables. The standard values of *R*^2^ are 0.75, 0.50, and 0.25 for high, medium, and low predictive accuracy, respectively (Hair et al., 2011). The *R*^2^ of the hypothetical model is 0.799, indicating high accuracy of control, organizational, and individual psychological factors on the Intention of Remaining.

In this study, the impact of the demographic variables (control variables), organization, and individual psychological factors on the Intention of Remaining are put into the hypothetical model at the same time. The above analysis shows that both the inner and outer models of the hypothetical model are relevant. Therefore, further analysis of the path coefficients of all the influencing factors will reveal which item has the highest influence on the Intention of Remaining.

#### Path Coefficient

**Figure 2:**
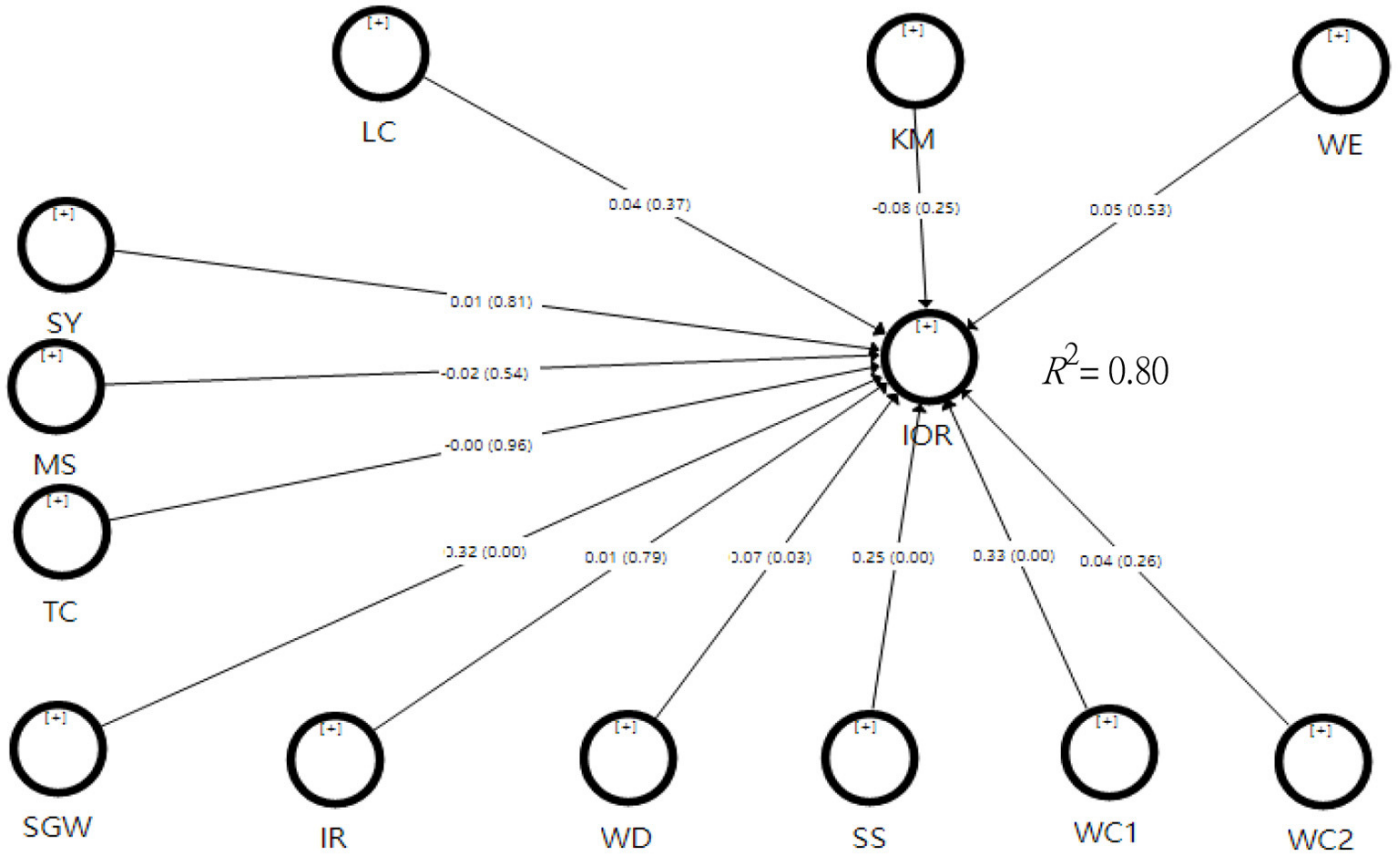
Test results of the overall model.

Concerning the path coefficients of the three demographic variables on the Intention of Remaining, the three paths of marriage (β = −0.018, *t* = 0.596, *p* = 0.551), service year (β = 0.007, *t* = 0.240, *p* = 0.81), and Teacher certificates (β = −0.001, *t* = 0.048, *p* = 0.961) did not reach the significant levels (see Figure 2). As for the path coefficients of organizational factors on the Intention of Remaining, the three paths of leadership characteristics (β = 0.036, *t* = 0.857, *p* = 0.392), kindergarten management (β = −0.075, *t* = 1.145, *p* = 0.252), and work environment (β = 0.052, *t* = 0.613, *p* = 0.54) did not reach the significant levels.

Regarding the path coefficients of individual psychological factors on the Intention of Remaining, the two paths of interpersonal relationships (β = 0.014, *t* = 0.273, *p* = 0.785) and work convenience (β = 0.036, *t* = 1.149, *p* = 0.25) did not reach the significant levels. While four paths of sense of gain from work (β = 0.321, *t* = 0.643, *p* = 0.000), work discard (β = 0.072, *t* = 2.24, *p* = 0.025), satisfaction with the salary (β = 0.248, *t* = 0.817, *p* = 0.000), and work connection (β = 0.334, *t* = 7.767, *p* = 0.000) reached the significant levels.

As the above results showed, three paths of organizational factors did not reach the significant levels, while two paths as interpersonal relationships and work convenience of individual psychological factors did not reach the significant level, but the other four paths reached the significant level. In terms of β, the four paths that reached significance were work connection, sense of gain from work, satisfaction with the salary, and work discard.

### Comparison of the Impact of Organizational and Individual Psychological Factors on the Intention of Remaining

In this study, two models of organizational and individual psychological factors were independently analyzed, and the *R*^2^ of the two models was compared.

#### Organizational Factor Model

As the model was based solely on organizational factors and control variables, the relevance of the model had to be re-examined. The results showed that the relevance of the observed indicators ranged from 0.630 to 1.000, the combined relevance ranged from 0.958 to 1.000, the AVE ranged from 0.740 to 1.000, and the square root of AVE for each factor was greater than the correlation between the potential variable and the other variables. In conclusion, all four data met the criteria, indicating that the outer model was relevant. For the inner model, SRMR = 0.500, *Q*^2^ = 0.420, >0, with the criteria, and *R*^2^ =0.7 98 indicating its compliance and a high degree of predictive accuracy. In addition, the overall model had an *R*^2^ = 0.799, which decreased by 0.179 after removing three potential variables, such as organizational factors.

**Figure 3:**
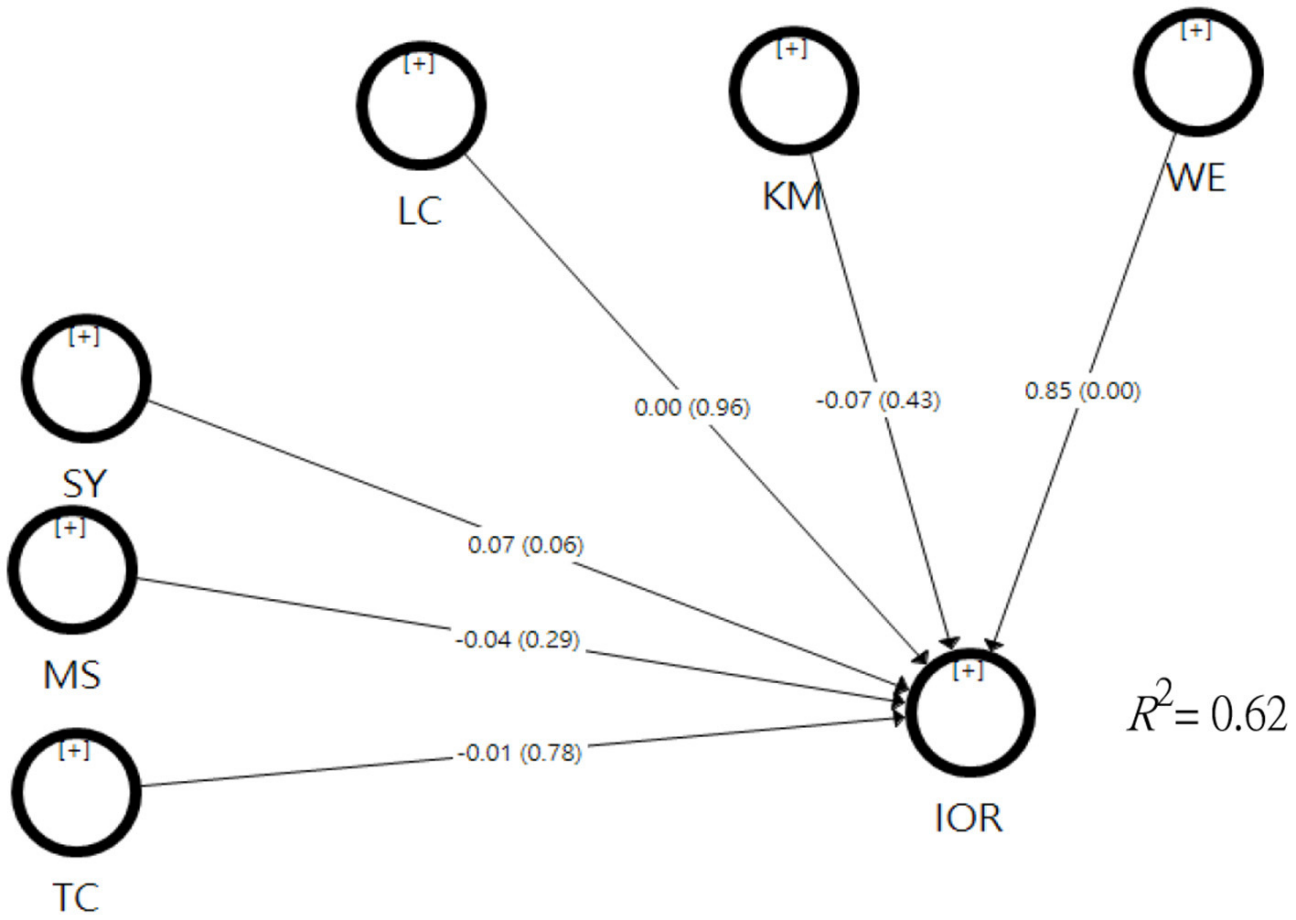
Test results of the organizational factors hypothesis model.

From the analysis of the relevance of outer and inner models, the model of organizational factors was relevant. Therefore, the impact can be understood by further comparing the path coefficients of individual factors. For the path coefficients, among leadership characteristics (β = 0.002, *t* = 0.043, *p* = 0.965), kindergarten management (β = −0.074, *t* = 0.804, *p* = 0.414), and work environment (β = 0.853, *t* = 10.392, *p* = 0.000), only work environment reached the significant levels (see Figure 3), indicating that it had the highest influence among the organizational factors.

#### Individual Psychological Factor Model

In terms of outer model fit, the indicator reliabilities ranged from 0.566 to 1.000, the combined reliabilities ranged from 0.905 to 1.000, and the AVE ranged from 0.656 to 1.000, with the square root of the AVE for each factor being greater than the value of the correlation between the latent variable and the other variables. In conclusion, all four data met the criteria and indicated that the measurement model was appropriate. For the inner model, SRMR = 0.500, *Q*^2^ = 0.546, and *R*^2^ = 0.798, indicating its compliance with the criteria and a high degree of predictive accuracy. In addition, the overall model had an *R*^2^ = 0.799, which decreased by 0.001 after removing three potential variables such as organizational factors.

**Figure 4:**
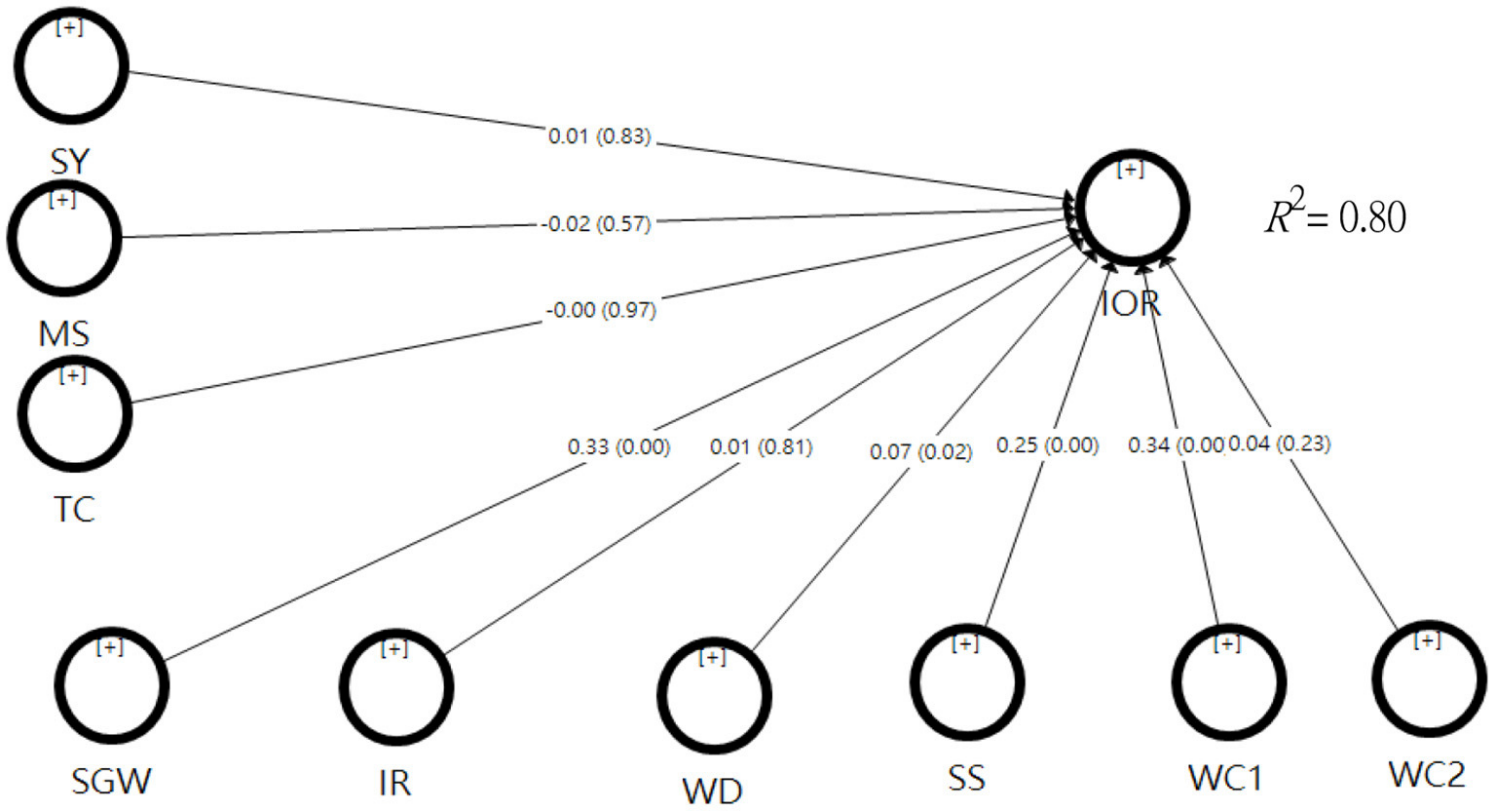
Test results of the individual psychological factors hypothesis model.

From the analysis of the relevance of outer and inner models, the model of the individual psychological factors was relevant. Therefore, the impact can be understood by further comparing the path coefficients of individual factors. The path coefficients, interpersonal relationships (β = 0.011, *t* = 0.237, *p* = 0.813), and work convenience (β = 0.038, *t* = 1.191, *p* = 0.234) did not reach the significant levels. Nevertheless, sense of gain from work (β = 0.327, *t* = 7.401, *p* = 0.000), work discard (β = 0.074, *t* = 0.306, *p* = 0.021), satisfaction with the salary (β = 0.247, *t* = 6.256, *p* = 0.000), and work connection (β = 0.337, *t* = 7.729, *p* = 0.000) reached the significant levels (see Figure 4).

#### Comparison of the Two Models

Combining the above analysis, the overall model has an *R*^2^ = 0.799, the organizational factor model has an *R*^2^ = 0.620 after removing the six potential variables, such as personal psychological factors, which is 0.179 lower, and the individual psychological factor model has an *R*^2^ = 0.798 after removing three potential variables, such as organizational factors, which is 0.001 lower. It can also be seen from the overall model that only four paths of individual psychological factors reach the significant level, but three paths of organizational factors do not. Thus, the impact of individual psychological factors on the Intention of Remaining is higher than that of the organizational factors.

## Discussion

The demographic variables (control variables, three factors), organizational factors (three factors), and individual psychological factors (six factors) are placed in a structural equation model based on variances, and the results show that both the outer and inner models are relevant, indicating that the overall model is relevant. And the overall model has an *R*^2^ = 0.799, indicating that the organizational and individual psychological factors are highly accurate on the Intention of Remaining after controlling the demographic variables. In other words, these factors explain 79.90% of Intention of Remaining. However, it should also be noted that the three paths of organizational factors leadership characteristics, kindergarten management, and work environment, and two paths of individual psychological factors interpersonal relationships and work convenience do not reach significant levels, indicating that these five factors did not have significant explanatory power in the overall model. In contrast, four paths as work connection, sense of gain from work, satisfaction with the salary, and work discard (sorted by β values) reach significant levels. Therefore, these four items are the important factors that influence the Intention of Remaining teachers in private kindergartens.

The three paths of organizational factors as leadership characteristics, kindergarten management, and work environment, and two paths of individual psychological factors interpersonal relationships and work convenience do not reach significant levels, and this result has a discrepancy with the existing studies. There are three possible reasons for this discrepancy. First, the subjects of the existing studies are mostly primary and secondary school teachers while the subjects of this study are teachers in private kindergartens, whose working characteristics are of great difference, so they cannot be compared. Second, teachers in private kindergartens are not state-funded but employed based on labor contracts, so their work stability is poor, leading to a lack of security. Although the information provided through the questionnaire is confidential, they may still have concerns when doing the questionnaire, thus being reluctant to reveal their true thoughts. They are afraid of subsequent retaliation if they put the responsibility of the factors affecting the Intention of Remaining on leadership characteristics, kindergarten management, and work environment, thus affecting the reliability of the results. Third, the subjects of this study are in-service teachers, who may be satisfied with everything and concerned less about the influencing factors that are important to them. Those who are not satisfied may have already turnover. Further relevant research needs to be strengthened these points subsequently.

Both organizational and individual psychological factor models are relevant in both the inner and outer models after controlling for the impact of demographic variables. The organizational factors have an *R*^2^ = 0.620 and individual psychological factors have an *R*^2^ = 0.798 under controlling the demographic variables. When subtracted from *R*^2^ of the overall model, *R*^2^ of the organizational factor model decreases by 0.179 and the individual psychological factor model by 0.001, which shows that the explanatory power of the individual psychological model is higher than that of the organizational model. As a result, the six paths of individual psychological factors have a higher influence on the Intention of Remaining teachers in private kindergartens than the organizational factors.

In addition, interpersonal relationships and work convenience do not reach significant levels in both the overall model and individual psychological factor model, while work connection, sense of gain from work, satisfaction with the salary, and work discard reached significant levels. The result is consistent with the ranking in the overall model. In other words, these four paths are important factors affecting the Intention of Remaining teachers in private kindergartens compared to others. These four factors are discussed as follows.

First, work connection refers to whether private kindergarten teachers are accustomed to their current work environment and status, whether they stay in kindergartens because of their sense of responsibility, whether they enjoy working with children, or whether they have a dream to improve the current situation of preschool education. In other words, the most powerful explanations for the Intention of Remaining related to the sense of responsibility, liking children, the dream of improving preschool education, and getting used to the current work. These are expressions of professional affective commitment of teachers in private kindergartens, which are teachers' identification and affection for their work and willingness to actively contribute to the kindergartens (Zhang, 2009).

Work connection is also a part of job embeddedness, which describes the organizational and community-related factors that keep employees in their jobs and make them attach or embed in their jobs, even when they are not satisfied with their current job and has opportunities for job-hopping (Mitchell and Lee, 2001; Mitchell et al., 2001). This study has discovered that when some teachers want to turnover in private preschools, but the children in their classes are not ready to graduate, quitting suddenly will be a sign of irresponsibility, which is consistent with the theory of job embeddedness. As a result, even when employees are dissatisfied with their jobs, they will choose to stay because of their attachment to the children and embeddedness in their current jobs. Job embeddedness is resistance to turnovers (Yang et al., 2014). Therefore, the sense of responsibility for their current job, their love for the children, their dreams to change preschool education, and their adaptation to their current job are all concrete manifestations of the Intention of Remaining teachers in private kindergartens.

Second, the sense of gain from work is an emotional experience and a perception of rewards, including a sense of happiness, accomplishment, belonging, fulfillment, presence, etc. for teachers in private kindergartens. If the perception is high, the Intention of Remaining private kindergarten teachers will be improved. Besides, if teachers can improve their knowledge and abilities, express their ideas and show their talents, and support their professional development (e.g., more training and learning opportunities) in private preschools, their Intention of Remaining will be improved. Otherwise, the limited space for professional development will lead to their turnovers (Duan, 2017).

Third, numerous studies related to factors influencing kindergarten teachers' turnover mentioned salaries. The study finds that satisfaction with the salary has significant explanatory power on the Intention of Remaining, which is consistent with the previous studies. For example, kindergarten teachers' satisfaction with salary affects the propensity of turnover, so the material dimension of salary is an important influencing factor on the turnover of teachers in private kindergartens (Fang and Deng, 2014; Duan, 2017). Therefore, salary is a double-edged sword, and a high salary can enhance the Intention of Remaining private kindergarten teachers.

One of the possible reasons for private kindergarten teachers for turnover and remaining is to evaluate their contributions and gains to see if they can accept their current salaries. Even if the overall salary is not high, they can accept it, and it will help to increase their Intention of Remaining. The second is to assess whether there is room for a raise, how much the raise will be, and how quickly a person can achieve it. If there is a chance of high raise within a short period, it will be attractive for the teachers and they will have a higher Intention of Remaining.

Fourth, work discard reflects private kindergarten teachers' perceptions of loss after turnover, such as the loss of leave and benefits that they have before; their worries about new jobs, and new teachers of their former classes, which are all concrete manifestations of unwillingness after turnover. The first four points reflect the perception of loss of personal benefits, but the last one reflects the teacher's emotional attachment to the children.

As for the reason why job choices affect the Intention of Remaining, it can be discussed from the topic that “I can find other jobs that have lower social status, reputation, and respect than kindergarten teachers.” Although the teachers in private preschools are not well paid overall, their social status and reputation are relatively high, which satisfies their spiritual dimension needs. Therefore, private kindergarten teachers' recognition that the job can have a higher social status, reputation, and respect is positive for their Intention of Remaining. In addition, “worries about the new teachers for their former classes” may indicate that teachers feel responsible for the children they teach and believe that the new teachers may not love the children as much as they do or provide as good a quality of care for the children as they do. It may also indicate that the overall quality of current private kindergarten teachers is low, and coupled with some incidences of child abuse, so their worries may be reinforced. Their worries about the new teachers show that the current private kindergarten teachers are dedicated and responsible to their work, as well as their attachment to their children. These emotions play a positive role in the Intention of Remaining teachers in mainland private kindergartens.

Work discard is closely related to the continuous commitment of organizations in connotation (a dimension of organizational commitment), which is manifested by employees' remaining because of concerns about the financial losses after a turnover or the obstacles posed by finding a new job (Zhang, 2009). Work discarded in this study reflects employees' perceptions of losses after turnover, and these perceptions hold the turnover of employees, i.e., they maintain remaining.

Finally, this study submits that a total of five paths do not reach significant levels, namely leadership characteristics, kindergarten management and work environment among organizational factors, and interpersonal relationships and work convenience among individual psychological factors. These findings differ from those of some studies, such as those by Fang et al. (2013), Guo (2014), Player et al. (2017), and Torres (2016), which confirmed that principals' modeling and the way they handle problems affect the teachers' Intention of Remaining. Organization justice and trust (Wang et al., 2014), organization fairness (Li et al., 2015), as well as organization support, can affect the employees' Intention of Remaining. However, according to Miller et al.'s (1999) study, teachers' perception of a hostile work environment is also an influencing factor for teachers' turnover. The reason for the inconsistency may lie in the different statistical methods, as these studies used product–moment correlation or multiple regressions for prediction instead of a comparison of all influencing factors in the same model. In contrast, this study uses a structural equation model, indirectly measuring the potential variables through the observed indicators and concluding the incorporation of errors into the calculation, which is more accurate (Chin, 1998), and allows observing the influence of potential variables on each other.

Previous research has shown that there are many factors that influence the intention to remain. For example, family factors (Huang et al., 2021), occupational factors (career commitment and job burnout) (Gaziel, 1995; Schaufeli and Taris, 2014; Huang et al., 2021), and emotional factors (emotional exhaustion and emotional labor) (Gao and Tan, 2017). This study considers too many questions in the questionnaire, which may affect the willingness to answer. Therefore, this study is limited to exploring the influence of organizational and personal psychological factors on the intention to remain.

Based on the research results, this paper has proposed some suggestions. From the perspective of government, great efforts should be made to protect the interest of the teachers and enhance the supervision and management of private kindergartens. At the level of kindergarten, teachers are vital for the development of the kindergarten. Various measures must be taken to strengthen their intention of remaining in the kindergartens. From the angle of teachers, they should make much deliberation about choosing their career and employment as well as make efforts to improve their professional abilities. As to the universities, they should select excellent students and cultivate excellent talents. Further efforts must be made to improve the education of preschool teachers and expand the employment opportunities.

## Conclusion

In this study, two questionnaires were developed and validated: the Influencing Factors of Private Kindergarten Teachers' Turnover questionnaire and the Influencing Factors of Private Kindergarten Teachers' Intention of Remaining questionnaire. Both questionnaires had good reliability and validity. The validation factor analysis revealed that the Private Kindergarten Teachers' Turnover questionnaire contained nine dimensions: LC, KM, WE, SGW, IR, WD, SS, WC1, and WC2. The absolute fit index, incremental fit measure, and parsimony fit index were significant, and the model fit was good. The impact of individual psychological factors on the kindergarten teachers' Intention of Remaining was greater than that of organizational factors.

## Data Availability Statement

The original contributions presented in the study are included in the article/supplementary material, further inquiries can be directed to the corresponding author/s.

## Author Contributions

ZZ and SS designed the study and wrote the protocol. HW and XZ carried out data analysis, collected data, and drafted the manuscript. ZZ conducted the critical revision. All authors contributed to the article and approved the submitted version.

## Funding

This study was supported by a grant 18CJY26 from the project of the Shandong Social Science Plan in 2018.

## Conflict of Interest

The authors declare that the research was conducted in the absence of any commercial or financial relationships that could be construed as a potential conflict of interest.

## Publisher's Note

All claims expressed in this article are solely those of the authors and do not necessarily represent those of their affiliated organizations, or those of the publisher, the editors and the reviewers. Any product that may be evaluated in this article, or claim that may be made by its manufacturer, is not guaranteed or endorsed by the publisher.
